# Scientific and Societal Considerations in Selecting Assessment Endpoints for Environmental Decision Making

**DOI:** 10.1100/tsw.2002.168

**Published:** 2002-03-08

**Authors:** Elizabeth M. Strange, Joshua Lipton, Douglas Beltman, Blaine D. Snyder

**Affiliations:** Stratus Inc, Boulder, CO 80306-4059, USA; Tetra Tech Inc., 10045 Red Run Blvd., Suite 110, Owings Mills, MD 21117, USA

**Keywords:** ecological risk assessment, assessment endpoints, measurement endpoints, population assessment, natural resource value, environmental value

## Abstract

It is sometimes argued that, from an ecological point of view, population-, community-, and ecosystem-level endpoints are more relevant than individual-level endpoints for assessing the risks posed by human activities to the sustainability of natural resources. Yet society values amenities provided by natural resources that are not necessarily evaluated or protected by assessment tools that focus on higher levels of biological organization. For example, human-caused stressors can adversely affect recreational opportunities that are valued by society even in the absence of detectable population-level reductions in biota. If protective measures are not initiated until effects at higher levels of biological organization are apparent, natural resources that are ecologically important or highly valued by the public may not be adequately protected. Thus, environmental decision makers should consider both scientific and societal factors in selecting endpoints for ecological risk assessments. At the same time, it is important to clearly distinguish the role of scientists, which is to evaluate ecological effects, from the role of policy makers, which is to determine how to address the uncertainty in scientific assessment in making environmental decisions and to judge what effects are adverse based on societal values and policy goals.

## INTRODUCTION

Ecological risk assessment is a process for evaluating the likelihood of adverse ecological effects[1,2]. It is designed to provide environmental decision makers with a scientific evaluation of the risks posed to ecological resources by alternative management actions, ranging from the regulation of hazardous waste sites to the management of entire watersheds affected by multiple stressors.

A critical component of the risk assessment process is the selection of assessment and measurement endpoints. Assessment endpoints are the environmental entities that are targets of the risk assessment, and measurement endpoints are the attributes that are actually measured[1,2]. For example, the reproductive success of Coho salmon is an assessment endpoint, while egg survival is a measurement endpoint.

Although numerous documents provide guidelines for endpoint selection[1,2], there remains some confusion about the role of science in the process. Some investigators argue that, from a scientific point of view, population- and higher-level endpoints should take precedence based solely on their ecological relevance[3,4,5]. However, as the EPA's ecological risk assessment guidelines make clear, scientific considerations are only part of the overall process of endpoint selection[2]. In many cases, social, economic, and policy considerations argue for the assessment of individual-level endpoints, as is the case for legally protected habitats or organisms, such as endangered species[6].

Even from a scientific perspective, there are compelling reasons for concluding that higher-level endpoints are not always appropriate or sufficient for assessing ecological risks. Whereas the measurement of higher-level endpoints may provide information about ecological condition, it may provide little information about the causes of observed effects. In contrast, individual-level endpoints are often preferred for ease and reliability of measurement and their relatively high statistical power to detect effects[7,8]. Moreover, individual effects are precursors to population and ecosystem effects, and thus individual-level effects help inform risk managers about potential future risks to higher levels of biological organization.

In this paper, we consider how endpoint selection is constrained by the need to balance ecological and management relevance with measurement validity and practicality, including the amount of time and money needed to complete a scientifically valid study. We outline key scientific, social, and policy considerations in the selection of endpoints and discuss some reasons why individual-level endpoints are sometimes preferable. We conclude by proposing that it is important to consider all of these factors to ensure that the risk assessment process will support the overall goal of environmental protection.

## SCIENTIFIC CONSIDERATIONS IN SELECTING RISK ASSESSMENT ENDPOINTS

According to the EPA's *Guidelines for Ecological Risk Assessment*, selection of assessment endpoints should consider (1) susceptibility to the stressor, (2) ecological relevance, and (3) policy goals and societal values[2]. In this section, we consider issues related to ecological relevance.

Although important for evaluating overall ecological condition, there can be ambiguity and uncertainty in population-, community-, and ecosystem-level assessments resulting from natural variability, measurement difficulties, lack of data, and limitations of scientific understanding[9].

Detection of higher-level effects is difficult in large part because of the natural variation inherent in biological populations[7,8]. For example, studies show that it can take at least a decade or two to detect a “signal” from the “noise” in fish population data[10]. Natural variation also means that it is often difficult to establish “baseline” or “average” conditions against which the significance of impacts can be evaluated[7,8,11]. Long-term monitoring can help reduce uncertainties, but this is costly and impractical in many contexts[9,12].

Cause-effect relationships are also difficult to establish at higher levels of biological organization[13], although the stressor identification process has advanced in recent years[14]. Populations, communities, and ecosystems reflect effects of multiple stressors interacting in complex ways[15]. Characteristics of these entities integrate all stressor effects, and therefore it can be very difficult to attribute population- or higher-level ecological effects to any particular stressor. For example, distinguishing the relative impacts of various environmental stressors on declines of salmon (*Oncorhynchus* spp.) in the Pacific Northwest, lake trout (*Salvelinus namaycush*) in the Great Lakes, and many other fish species has proven to be very difficult despite years of study by numerous researchers[16].

Defining the spatial and temporal boundaries of higher-level ecological entities is also difficult and often arbitrary[17]. For example, a fish population can be defined on the basis of the local stock or in terms of its regional extent. Mortalities of individuals may significantly reduce the local population, while effects on the regional population may remain undetectable.

A prominent example of conflicts over population-level impacts has been the ongoing debate over the impacts on fish populations caused by larval entrainment in the cooling water intakes of power plants[18,19]. Most assessments of power plant entrainment have been based on population models with significant uncertainties, such as the potential role of density-dependent compensation in response to power plant mortality. As a result, there has been little agreement about whether or not adverse impacts are occurring, despite the enormous losses of aquatic organisms at power plant intakes.

There is much less uncertainty in individual-level assessments[20]. In most cases, individuals can be defined with less ambiguity and greater ease. Measurement and sampling errors at the individual level are also less than those associated with estimates of populations[7,8]. As a result of greater data availability and reliability, environmental effects are more likely to be detected at the individual level than at higher levels of biological organization.

For example, Bennett et al. [21] found a high percentage of abnormalities in larval striped bass that were thought to result from herbicide use in rice fields, as indicated by the absence of abnormalities following changes in culture practices that reduced herbicide release into rivers with striped bass. In addition, Bailey et al.[22]

| Entity | Response Time | Ambiguity | Difficulty of Measurement | Relevance for Ecosystem |
|---|---|---|---|---|
| individual | short | low | low | less direct |
| population | | | | |
| community | | | | |
| ecosystem | long | high | high | more direct |

**FIGURE 1.** Tradeoffs in endpoint selection**.**

found that the decline of striped bass in California was correlated with increased herbicide use. Nevertheless, Kimmerer et al.[23] could find no evidence of a population-level response.

Environmental decision makers must often balance the need for ecological relevance with the need for measurement ease and reliability in deciding what endpoints to evaluate (Fig. 1). In cases where a stressor directly affects individuals, but population or higher-level effects are unclear though potentially important, individual-level endpoints may need to take precedence. Indeed, effects on individuals can be important predictors of potential effects on populations or communities that cannot be measured directly.

## The Role of Social Values and Policy Goals in Endpoint Selection

While scientific considerations are important, they are not the only factors that environmental decision makers must take into account in evaluating the potential for adverse effects. In fact, the EPA's *Ecological Risk Assessment Guidelines* stress that the appropriate level of biological organization for an assessment depends on societal values and policy goals as well as data availability and ecological relevance[2]. Indeed, society clearly places value on ecological attributes that are not necessarily captured by assessing only higher levels of biological organization, and thus individuals may warrant protection even *in lieu* of population-level effects.

For example, a survey following the Nestucca oil spill in the state of Washington found that local residents believed that preventing the death of seabirds from oil spills is important, even if seabird populations appear unaffected[24].

Similarly, in a regional survey conducted as part of a natural resource damage assessment for Green Bay, people expressed high value (hundreds of millions of dollars) for restoring bird and fish injuries from PCBs, even though they were explicitly told that there may not be population-level effects[25].

## Regulatory Guidance

The value that society places on individual organisms is reflected in many current regulations and statutes. As described below, the Clean Water Act (CWA), the Migratory Bird Treaty Act, the Comprehensive Environmental Response, Compensation and Liability Act (CERCLA), the Oil Pollution Control Act (OPA), the Federal Insecticide, Fungicide and Rodenticide Act (FIFRA), and relevant case law authorize that effects at the individual organism level be assessed in making regulatory decisions.

In some cases, risk assessments and regulatory programs consider effects on individuals to be important as indicators of effects on populations. In these cases, individual-level effects are a measurement endpoint for the population, which is the assessment endpoint. An example is provided by the National Pollution Discharge Elimination System (NPDES) permit program. Under section 301(b)(1)(c) of the CWA, effluent limits must be placed in NPDES permits as necessary to meet water quality standards. To implement this requirement, the EPA and most states rely on toxicity tests that determine the effects of discharges on individual organisms[26]. By evaluating the effects of pollutants on growth, reproduction, and mortality of individuals, the EPA uses individual impacts as surrogates and precursors of population and ecosystem impacts.

In other cases, risk assessments and regulatory programs are intended to protect individual members of a species, regardless of potential effects on the population of the species. For example, the Migratory Bird Treaty Act, 16 U.S.C. §§ 703-712, prohibits, among other things, the killing of individual migratory birds [16 U.S.C. §703]. The act does not require evidence that bird mortalities affect a bird population; effects on individual organisms are the only test.

Another example is provided by CERCLA [42 U.S.C. Section 9601 *et seq*.] and OPA [33 U.S.C. Section 2701 *et seq*.], which require that the public be compensated for natural resource injuries resulting from an oil spill or hazardous substance release. These regulations stipulate that the value of lost resources can include the value of injured individuals of marine species as well as the value that society places on just knowing that a natural area exists.

A final example of regulations designed to protect individuals is provided by FIFRA, 7 U.S.C., which regulates the manufacture, distribution, and use of pesticides. The act is intended to protect the "water, air, land, and all plants and man and other animals living therein, and the interrelationships which exist among these" [7 U.S.C. §136 (j)] from unreasonable adverse effects [7 U.S.C. §136 (d)]. Under FIFRA, effects on biological populations are not a required element of risk assessment. A 1989 decision by the U.S. Court of Appeals for the Fifth Circuit illuminates how "unreasonable adverse effects" are interpreted under FIFRA. In 1988, the EPA canceled registration for the pesticide diazinon unless registration was amended to prohibit use on golf courses and sod farms, based on the EPA's determination that the use of the pesticide in these cases posed an unreasonable risk to birds [53 Fed. Reg. 11119].

Ciba-Geigy Corporation, diazinon's major producer, petitioned the EPA's determination for review by the courts. Among other issues, Ciba-Geigy presented the argument that a risk is unreasonable only if it endangers bird populations, not just individuals [55 Fed. Reg. 31137]. The court rejected Ciba-Geigy's argument, stating that "FIFRA gives the Administrator sufficient discretion to determine that recurring bird kills, even if they do not significantly reduce bird populations, are themselves an unreasonable environmental effect" [874 F.2d 277].

The court clearly sided with the EPA in its determination that effects at the individual organism level can be interpreted as unreasonable environmental effects.

## Risk Assessment in the Overall Context of Environmental Decision Making

Current guidelines by the EPA and other environmental agencies indicate that whether estimated risks are considered "adverse," "undesirable," or "unacceptable" should be based on a range of factors, including management goals, policy considerations, societal values, and legal mandates, as well as underlying scientific understanding[2]. Thus, there is no universal definition of "adverse environmental impact," nor can there be. Ultimately, the decision of what is "adverse" rests with policy makers, not scientists. As Rykiel[27] noted: "... science deals with true and false, whereas society deals with good and bad." While someone must decide what ecological conditions are good or bad, it should not be scientists if we are to maintain scientific impartiality[28,29].

Environmental decision makers face a difficult task in choosing from among what are often competing social values. Even cost-benefit comparisons of management options provide few clear-cut answers. As Lackey[29] pointed out: "The marketplace, the most common adjudicator of societal preferences, is never totally unconstrained, nor do most participants have much understanding of the long-term ecological consequences of their individual market decisions. Thus, economics has an important role in resolving competing societal preferences, but is insufficient in itself." Moreover, many biological resources that are valued by society are not traded in markets, and failure to account for these assets can seriously bias environmental decision making[30].

When individual-level effects are considered, the regulatory scope for minimizing impacts to environmental resources is greater than it is for minimizing higher-level impacts. This is because individual effects are more likely to be detected. A focus on the most readily detected effects allows risk managers to undertake actions to reduce impacts before more serious damage to higher levels of organization can occur.

Many resource agencies recognize that if protective measures are not initiated until effects at higher levels of biological organization are apparent, natural resources that are ecologically important or highly valued by society may not be adequately protected. This has led these agencies to exercise a “precautionary approach” to environmental management[31]. The precautionary approach aims to prevent irreversible damage to the environment by implementing strict conservation measures even in the absence of unambiguous scientific evidence that environmental degradation is being caused by human stressors[32].

The precautionary approach is now being applied in fisheries management. For example, in a recent publication, the National Marine Fisheries Service (NMFS) noted that “all fishing activities have environmental impacts and that it is not appropriate to assume that these impacts are unimportant until proven otherwise[31].” The report concluded that the collapse of fish stocks worldwide has resulted in part because corrective actions were often delayed or not implemented when scientific information on stock status was in doubt. NMFS noted that, in 1995, the Food and Agriculture Organization (FAO) of the United Nations drafted an International Code of Conduct that emphasized that “the absence of adequate scientific information should not be used as a reason for postponing or failing to take conservation management measures.”[31]

## CONCLUSIONS

While the purpose of an ecological risk assessment is to provide environmental decision makers with a scientific evaluation of the risks posed to ecological resources, science cannot answer the difficult question of how much impact is acceptable[29,33,34,35,36]. The distinction between the role of scientists in evaluating ecological effects and the role of policy makers in judging the adversity of effects is important, but often overlooked. To avoid unnecessary conflicts, it is critical to clearly separate the roles of scientists and policy makers in the risk assessment process. Failure to do so may not only undermine the objectivity necessary for valid risk assessment, but can ultimately interfere with the overriding goal of environmental protection.

## ACKNOWLEDGEMENTS

Support for this work was provided, in part, by the U.S. EPA to Stratus Consulting Inc. under Contract No. 68-W6-0055 and to Tetra Tech under Contract No. 68-C-99-249. However, the views expressed in this paper are those of the individual authors, and do not represent the official position of the U.S. EPA. The authors wish to thank John Boreman, James Andreason, and Peter Moyle for their helpful comments and suggestions on an earlier draft of this manuscript.

## References

1. Suter, G.W. (1993) *Ecological Risk Assessment*. Lewis Publishers, Chelsea, MI.
2. U.S. EPA (1998) Guidelines for Ecological Risk Assessment. EPA/630/R-95/002B. U.S. Environmental Protection Agency, Washington, D.C.

3. Clements, W.H. and Kiffney, P.M. (1994). Assessing contaminant effects at higher levels of biological organisation. *Envron. Toxicol. Chem.* **13**, 357–359.
4. Martin, M. and Richardson, B.J. (1995) A paradigm for integrated marine toxicity research? Further views from the Pacific Rim. *Mar. Pollut. Bull.* **30**, 8–13.
5. Dey, W.P., Jinks, S.M., and Lauer, G.J. (2000) The 316(b) assessment process: evolution towards a risk-based approach. *Environ. Sci. Policy* **3**, S15–S23.
6. Schmitt, R.J., Osenberg, C.W., Douros, W.J., and Chesson, J. (1996) The art and science of administrative environmental impact assessment. In *Detecting Ecological Impacts: Concepts and Applications in Coastal Habitats.* Schmitt, R.J. and Osenberg, C.W., Eds. Academic Press, Inc., San Diego, CA. pp. 281–293.
7. Osenberg, C.W., Schmitt, R.J., Holbrook, S.J., Abu-Saba, K.E., and Flegal, A.R. (1994) Detection of environmental impacts: natural variability, effect size, and power analysis. *Ecol. Appl.* **4**, 16–30.
8. Osenberg, C.W., Schmitt, R.J., Holbrook, S.J., Abu-Saba, K.E., and Flegal, A.R. (1996) Detection of environmental impacts: natural variability, effect size, and power analysis. In *Detecting Ecological Impacts: Concepts and Applications in Coastal Habitats.* Schmitt, R.J. and Osenberg, C.W., Eds. Academic Press, Inc., San Diego, CA. pp. 83–108..
9. Schmitt, R.J. and Osenberg, C.W., Eds. (1996*) Detecting Ecological Impacts: Concepts and Applications in Coastal Habitats.* Academic Press, San Diego, CA.
10. Myers, R.A., Bridson, J., and Barrowman, N.J. (1995) Summary of worldwide stock and recruitment data. *Can. Tech. Rep. Fish. Aquat. Sci.* **2024**, 1–327.
11. Stewart-Oaten, A. (1996) Problems in the analysis of environmental monitoring data. In *Detecting Ecological Impacts: Concepts and Applications in Coastal Habitats*. Schmitt, R.J. and Osenberg, C.W., Eds. Academic Press, Inc., San Diego, CA. pp. 109–132.
12. NRC (National Research Council) (1990) Managing Troubled Waters: The Role of Marine Environmental Monitoring. National Academy Press, Washington, D.C.
13. Attrill, M.J. and Depledge, M.H. (1997) Community and population indicators of ecosystem health: targeting links between levels of biological organization. *Aquat. Toxicol.* **38**, 183–197.
14. U.S. EPA (2000) Stressor Identification Guidance Document. EPA/822/B-00/025, U.S. Environmental Protection Agency, Office of Water and Office of Research and Development, Washington, D.C.
15. U.S. EPA (1992) Biological Populations as Indicators of Environmental Change. EPA-230-R-92-011, U.S. Environmental Protection Agency, Office of Policy Planning and Evaluation, Washington, D.C.
16. Walters, C. (1997) Challenges in adaptive management of riparian and coastal ecosystems. *Conserv. Ecol.* [online] **1**,1–23. URL:http://www.consecol.org/vol1/iss2/art1
17. Levin, S.A. (1992) The problem of pattern and scale in ecology. *Ecology* **73**, 1,943–1,976.
18. Christensen, S.W., Van Winkle, W., Barnthouse, L.W., and Vaughan, D.S. (1981) Science and the law: confluence and conflict on the Hudson River. *Environ. Impact Assess. Rev.* **2/1**, 63–88.
19. Barnthouse, L.W., Klauda, R.J., Vaughan, D.S., and Kendall, R.L., Eds. (1988) Science, law, and Hudson river power plants: a case study in environmental impact assessment. *Am. Fish. Soc. Monogr*. **4**.
20. DeAngelis, D.L., Barnthouse, L.W., Van Winkle, W., and Otto, R.G. (1990) A critical appraisal of population approaches in assessing fish community health. *J. Great Lakes Res.* **16**, 576–590.
21. Bennett, W.A., Ostrach, D.J., and Hinton, D.E. (1995) Larval striped bass condition in a drought-stricken estuary: evaluating pelagic food-web limitation. *Ecol. Appl.* **5,** 680–692.
22. Bailey, H.C., Alexander, C., Digiorgio, C., Miller, M., Doroshov, S.I., and Hinton, D.E. (1994) The effect of agricultural discharge on striped bass (*Morone saxatilis*) in California's Sacramento-San Joaquin drainage. *Ecotoxicology* **3**, 123–142.
23. Kimmerer, W.J., Cowan, Jr., J.H., Miller, L.W., and Rose, K.A. (2000) Analysis of an estuarine striped bass (*Morone saxatilis*) population: influence of density-dependent mortality between metamorphosis and recruitment. *Can. J. Fish. Aquat. Sci.* **57**, 478–486.
24. Rowe, R.D., Schulze, W.D., Shaw, W.D., Schenk, D., and Chestnut, L.G. (1991) Contingent valuation of natural resource damage due to the Nestucca Oil Spill. Final Report prepared for Department of Wildlife, State of Washington, Olympia, WA; British Columbia Ministry of Environment, Victoria, BC; Environment Canada, Vancouver, BC, Canada.

25. Breffle, W.S., and Rowe, R.D. (2002) Comparing choice question formats for evaluating natural resource tradeoffs. *Land Econ.,* in press.
26. U.S. EPA (1991) Technical Support Document for Water Quality-Based Toxics Control. EPA/505/2-90-001. U.S. Environmental Protection Agency, Washington, D.C.
27. Rykiel, E.J. (1998) Relationships of scale to policy and decision making. In *Ecological Scale: Theory and Applications*. Peterson, D.L. and Parker, V.T., Eds. Columbia University Press, New York. pp 485–497.
28. Sagoff, M. (1995) The value of integrity. In *Perspectives on Ecological Integrity*. Westra, L. and Lemons, J., Eds. Kluwer Academic Publishers, Dordrecht, The Netherlands. pp. 162–176.
29. Lackey, R.T. (2001) Values, policy, and ecosystem health. *BioScience* **51**, 437–443.
30. Lipton, D.W., Wellman, K., Sheifer, C., and Weiher, R.F. (1995) Economic valuation of natural resources — a handbook for coastal resource policymakers. NOAA Coastal Ocean Program Decision Analysis Series No. 5. NOAA Coastal Ocean Office, Silver Spring, MD, 131 p.
31. National Marine Fisheries Service (1999) The precautionary approach: a new paradigm or business as usual? Our Living Oceans. Report on the Status of U.S. Living Marine Resources. U.S. Department of Commerce, NOAA Tech. Memo. NMFS-F/SPO-41. pp. 61–70.
32. Hilborn, R., Maguire, J.-J., Parma, A.M., and Rosenberg, A.A. (2001) The precautionary approach and risk management: can they increase the probability of success in fishery management? *Can. J. Fish. Aquat. Sci.* **58**, 99–107.
33. Salzman, L. (1995) Scientists and advocacy. *Conserv. Biol.* **9**, 709–710.
34. Lackey, R.T. (1998) Seven pillars of ecosystem management. *Lands. Urban Plan*. **40**, 21–30.
35. Lackey, R.T. (1999) The savvy salmon technocrat: life's little rules. *Environ. Pract.* **1**, 156–161.
36. Power, M. and McCarty, L.S. (1997) Fallacies in ecological risk assessment practices. *Environ. Sci. Technol*. **31**, 370A–375A.

## BIOSKETCH

**Elizabeth M. Strange** is a Manager at Stratus Consulting Inc., an environmental and energy research firm in Boulder, Colorado. Dr. Strange is an aquatic ecologist with expertise in the assessment of human impacts to marine and freshwater ecosystems. She has developed and assessed ecological endpoints for quantifying benefits of proposed regulations, assessing resource injuries, comparing restoration options, and predicting potential consequences of climate change and other global stressors on aquatic ecosystem services. Her work has included the collection, analysis, and modeling of fisheries and water quality data for regulatory impact assessments and natural resource damage assessments. Dr. Strange has also worked closely with natural resource economists to develop methods for integrating environmental assessments and benefits estimation. She has published results of her research in a number of peer-reviewed journals, including *Environmental Management*, *Ecological Economics*, *Environmental Biology of Fishes*, and *Marine Fisheries Review*. Dr. Strange holds a Ph.D. and an M.S. in ecology from the University of California at Davis and a B.A. in biology from San Francisco State University.
